# Long-Term Prognostic Impact of Three-Dimensional Speckle-Tracking Echocardiography-Derived Left Ventricular Global Longitudinal Strain in Healthy Adults—Insights from the MAGYAR-Healthy Study

**DOI:** 10.3390/jcdd11080237

**Published:** 2024-07-31

**Authors:** Attila Nemes, Árpád Kormányos, Dorottya Lilla Olajos, Alexandru Achim, Zoltán Ruzsa, Nóra Ambrus, Csaba Lengyel

**Affiliations:** Department of Medicine, Albert Szent-Györgyi Medical School, University of Szeged, H-6720 Szeged, Hungary; kormanyos.arpad@med.u-szeged.hu (Á.K.); olajosd@gmail.com (D.L.O.); zruzsa25@gmail.com (Z.R.); ambrusnora@gmail.com (N.A.); lecs@in1st.szote.u-szeged.hu (C.L.)

**Keywords:** global longitudinal strain, speckle-tracking, prognosis, healthy

## Abstract

Introduction. Three-dimensional (3D) speckle-tracking echocardiography (STE) combines the advantages of STE and volumetric 3D echocardiography, which shows the left ventricle (LV) in 3D during the cardiac cycle and is also suitable for accurate strain measurements in addition to volumetric assessments using the same virtual 3D LV cast. The present study aimed to confirm the prognostic impact of 3DSTE-derived LV global longitudinal strain (GLS) in healthy adults during a 12-year follow-up period. Patients and methods. The current study comprised 124 healthy individuals with a mean age of 31.0 ± 11.7 years (64 males) at the time of complete two-dimensional Doppler echocardiography (2DE) and 3DSTE. Results. During a mean follow-up of 8.01 ± 4.12 years, 10 healthy individuals suffered cardiovascular events, including 2 cardiac deaths. Using ROC analysis, 3DSTE-derived LV-GLS ≥ 14.77% was found to be a significant predictor for cardiovascular event-free survival (sensitivity 70%, specificity 71%, area under the curve 76%, *p* = 0.007). Using 2DE, higher LV end-diastolic and end-systolic volumes, a larger LV end-systolic diameter and a lower LV ejection fraction could be detected in subjects with LV-GLS < 14.77% as compared to cases with LV-GLS ≥ 14.77%. Subjects with events had thicker interventricular septa, a larger LV mass and lower 3DSTE-derived LV-GLS and a higher ratio of cases had LV-GLS < 14.77%. From subjects with LV-GLS < 14.77%, seven individuals (18%) had events. Multivariate regression analysis identified age and LV-GLS as independent predictors of event-free survival. Conclusions. 3DSTE-derived LV-GLS is a strong independent predictor of cardiovascular survival in healthy adults.

## 1. Introduction

The exact measurement of left ventricular (LV) function is of fundamental importance in judging the fate of patients. LV ejection fraction (EF), which is known and widely used in clinical practise, not only assesses the pumping function of the LV accurately but has a pronounced proven prognostic power as well [1,2]. Moreover, almost all cardiovascular imaging methods are capable of assessing LV-EF exactly, including M-mode, two-dimensional (2D), 2D speckle-tracking (STE), volumetric three-dimensional (3D) echocardiography and 3DSTE methods [1,3]. In addition to all these advantages, the assessment of LV-EF also has limitations, as its measurements are known to have a relatively high variability and reproducibility and it is load-dependent, which may limit its clinical significance [1,3,4].

With the spread of modern imaging methods, the so-called strains used for the quantitative characterisation of myocardial wall contractility have become a widely used testing method in recent decades [5]. In routine clinical practise, 2D-STE-derived LV global longitudinal strain (GLS) defined in a given plane is primarily used, which is an easy-to-implement and widespread method without load dependency but with proven normal reference ranges and well-documented prognostic power [1,6,7]. However, 2D-STE is based on certain assumptions about LV geometry; thus, echocardiographic examination based on virtually created 3D models of the given LV enables a more accurate analysis [1,8,9,10,11,12]. The 3DSTE method, now available in the clinical routine, combines the advantages of STE and volumetric 3D echocardiography, allowing LV volumetric and strain analysis in the 3D space with virtual models depicting the cardiac cycle using the same virtual 3D cast [3,8,9,10,11,12].

The prognostic impact of both 2D-STE- and 3DSTE-derived LV-GLS has been confirmed in certain pathological scenarios [7,13,14,15,16,17]. However, their prognostic significance in healthy circumstances has never been confirmed. Therefore, the present study aimed to confirm the prognostic value of 3DSTE-derived LV-GLS in healthy adults during a 12-year follow-up period.

## 2. Materials and Methods

### 2.1. Subjects

The current study comprised 124 healthy individuals with a mean age of 31.0 ± 11.7 years (64 males). All of them participated in the study on a voluntary basis and were recruited for screening between 2011 and 2015. In the case of all subjects, physical and laboratory tests, standard 12-lead electrocardiography (ECG) and 2D Doppler echocardiography were performed when findings proved to be within the normal reference ranges. None of the subjects had any known disease or pathological state or were obese or athletes, which could have an effect on the results. Medications or drugs were not used by any of the subjects. The present retrospective study is a part of the ‘Motion Analysis of the heart and Great vessels by three-dimensional speckle-tracking echocardiography in Healthy subjects’ (MAGYAR-Healthy Study) project, which was organized for several purposes, including to confirm the prognostic impact of certain 3DSTE-derived variables (‘Magyar’ means ‘Hungarian’ in the Hungarian language). The Institutional and Regional Human Biomedical Research Committee of University of Szeged, Hungary (No.: 71/2011), approved the study, which was conducted in accordance with the Declaration of Helsinki (as revised in 2013); all participants gave informed consent.

### 2.2. Follow-Up

The primary outcome of this study was cardiovascular mortality, including sudden cardiac death and hospitalization due to an invasive procedure, angina pectoris, acute heart failure, thrombosis or arrhythmia. All data were confirmed by medical recordings or autopsy reports.

### 2.3. Two-Dimensional Doppler Echocardiography

In all healthy individuals, the same commercially available cardiac ultrasound tool (Artida^TM^, Toshiba Medical Systems, Tokyo, Japan) attached to a broadband PST-30BT (1–5 MHz) phased-array transducer was used. With the healthy subject in the lateral decubitus position, grey-scale harmonic images and loops recorded on apical four- (AP4CH) and two-chamber (AP2CH) views were analysed and left atrial and LV dimensions, volumes and ejection fractions (EFs) were determined in accordance with the professional guidelines [1]. Valvular regurgitations and stenoses were determined with visual assessment using colour Doppler echocardiography.

### 2.4. Three-Dimensional Speckle-Tracking Echocardiography

**Figure 1:**
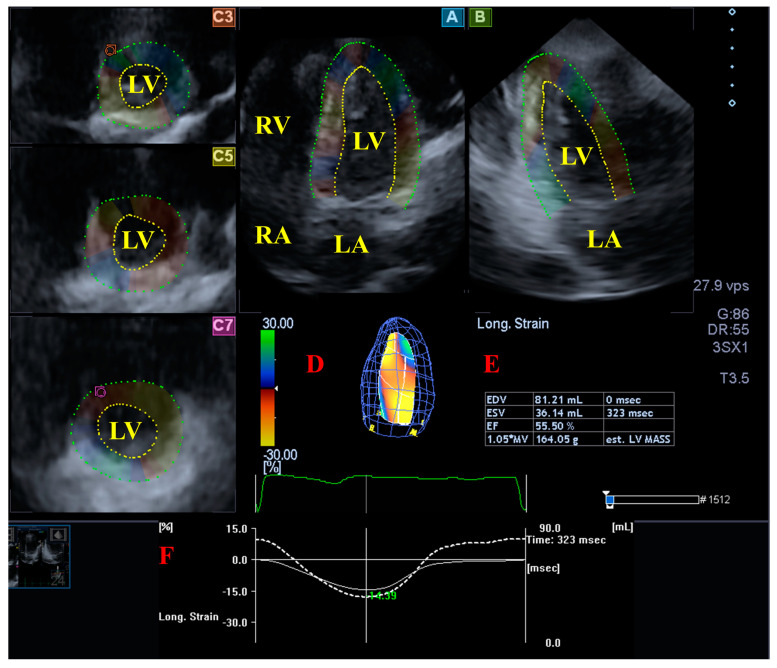
Analysis of the left ventricle (LV) from a three-dimensional (3D) speckle-tracking echocardiographic dataset: (A) apical four-chamber view, (B) apical two-chamber view and (C3) apical, (C5) midventricular and (C7) basal LV short-axis views. A virtual 3D cast of the LV (red D), LV volumetric data during the cardiac cycle (red E), time—LV global longitudinal strain (GLS, white line) and time—LV volume change (dashed white line) curves during the cardiac cycle (red F) are presented in a healthy subject. Abbreviations. LV = left ventricle, LA = left atrium, RV = right ventricle, RA = right atrium.

The 3DSTE examination was performed in 2 steps according to recent practices and guidelines [8,9,10,11]. First, 3D echocardiographic datasets were acquired using the same Toshiba Artida^TM^ (Toshiba Medical Systems, Tokyo, Japan) echocardiographic tool attached to another called a PST-25SX (1-4 MHz) matrix phased-array transducer. Data acquisition took place after image optimisation on gain, magnitude, etc. Six wedge-shaped subvolumes focused on LV were acquired within 6 consecutive heart cycles. The subject had to be on breath-hold and constant RR intervals were present on the ECG. The second step was realised at a later date offline when acquired full-volume 3D datasets were used for LV 3D quantifications using Wall Motion Tracking software version 2.7 (Toshiba Medical Systems, Tokyo, Japan). Each dataset was displayed in long-axis AP4CH and AP2CH views and in 3 short-axis views representing apical, midventricular and basal LV regions. The LV endocardium was manually defined at the lateral and septal edges of the mitral valve and the LV apex based on AP2CH and AP4CH views; then, a sequential analysis was performed by tracking the endocardium for anatomical reconstruction throughout the entire cardiac cycle to create a virtual 3D model of the LV. Then, LV-GLS was selected from the options offered by the software (Figure 1).

### 2.5. Statistical Analysis

All continuous variables were shown in the form of mean ± standard deviation, while categorical data were presented in the form of frequency/percentage (%). The Kolmogorov–Smirnov test was used to test the normality of the data. If they proved to have a normal distribution, Student’s *t*-test was used; if there was a non-normal distribution, the Mann–Whitney–Wilcoxon test was used. Fisher’s exact test was performed to analyse categorical variables. During the follow-up, survival of patients was assessed by Kaplan–Meier life table estimates. To test potential differences in survival rates between groups, a log-rank test was used. The prognostic power of LV-GLS was determined with receiver operator curves (ROCs) with sensitivity, specificity and area under the curve data. Statistical significance was considered in cases of *p* < 0.05 and all tests proved to be two-sided. Primary outcome was investigated by univariate analysis; the variables found to be significant (*p* < 0.10) were integrated into a Cox proportional hazard model as a multivariate approach with a forward stepwise model to analyse independent predictors of survival. For statistical analysis, SPSS software (version 22, SPSS Inc., Chicago, IL, USA) was used. Throughout the analysis, absolute values of LV-GLS were calculated with a positive sign.

## 3. Results

### 3.1. Clinical and Demographic Data

Clinical and demographic data are presented in Table 1. Body surface area (1.84 ± 0.13 kg/m^2^) and systolic (121.3 ± 3.6 mmHg) and diastolic (77.7 ± 2.9 mmHg) blood pressure values were in normal ranges. None of the subjects were smokers. By their own admission, none of them claimed to be regular drinkers. Subjects with events were older.

### 3.2. Events

During a mean follow-up of 8.01 ± 4.12 years, 10 healthy individuals suffered cardiovascular events, including 2 sudden cardiac deaths at the age of 33 and 54 (LV-EF: 57.1% and 53.8%, LV-GLS: 13.6% and 12.5%, respectively). Acute heart failure and angina pectoris developed in two patients (at the age of 60 and 70 with LV-EF: 55.4% and 50.6% and LV-GLS: 17.1% and 14.7%, respectively), three patients underwent invasive interventions (percutaneous coronary intervention with stent implantation at the age of 52, 55 and 58 with LV-EF: 60.5%, 48.7% and 51.6% and LV-GLS: 13.0%, 14.3% and 15.5%, respectively), two patients had deep vein thrombosis/pulmonary embolism at the age of 26 and 66 (LV-EF: 58.1% and 67.9% and LV-GLS: 13.5% and 12.9%, respectively), and one patient had an episode of paroxysmal supraventricular tachycardia at the age of 25 with LV-EF: 60.5% and LV-GLS: 15.7% (Table 1).

### 3.3. Left Ventricular Global Longitudinal Strain

**Figure 2:**
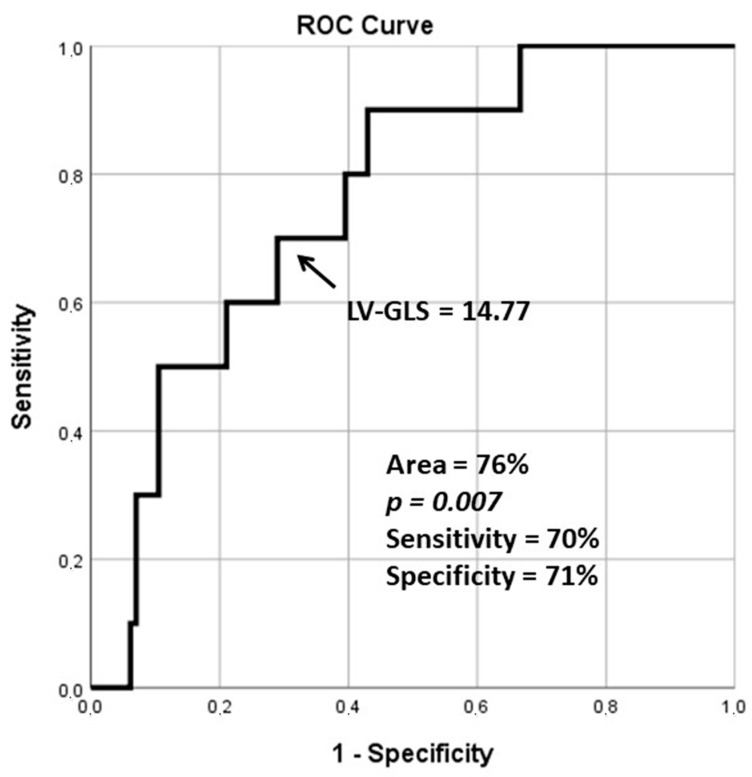
Receiver operating characteristic analysis illustrating the diagnostic accuracy of three-dimensional speckle-tracking echocardiography-derived left ventricular global longitudinal strain (GLS) in predicting cardiovascular morbidity and mortality.

**Figure 3:**
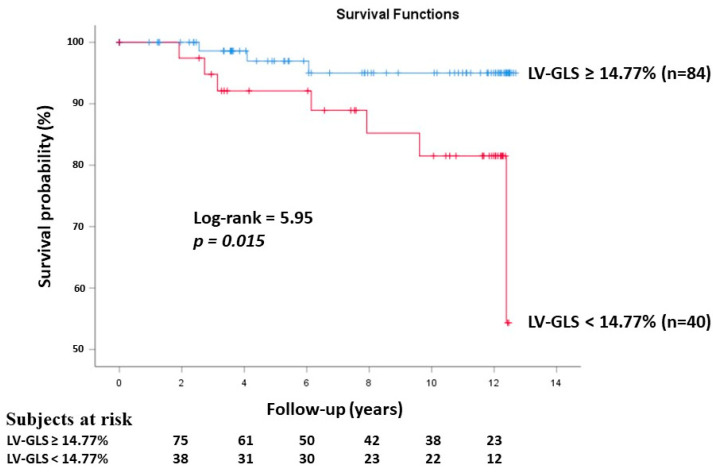
Kaplan–Meier survival curves illustrating the predictive role of three-dimensional speckle-tracking echocardiography-derived left ventricular global longitudinal strain (GLS).

Using ROC analysis, 3DSTE-derived LV-GLS ≥ 14.77% was a significant predictor for cardiovascular event-free survival (sensitivity 70%, specificity 71%, area under the curve 76%, *p* = 0.007) (Figure 2). The Kaplan–Meier cumulative survival curve illustrating the predictive role of 3DSTE-derived LV-GLS is presented in Figure 3.

### 3.4. Two-Dimensional Echocardiography

Routine 2D echocardiographic data are presented in Table 1. Higher LV end-diastolic and end-systolic volumes, larger LV end-systolic diameter and lower LV-EF values were detected in subjects with LV-GLS < 14.77% as compared to cases with LV-GLS ≥ 14.77%. Subjects with events had thicker interventricular septa.

### 3.5. Three-Dimensional Speckle-Tracking Echocardiography

The mean frame rate proved to be 31 ± 3 fps. Subjects with LV-GLS < 14.77% had more events as compared to cases with LV-GLS ≥ 14.77%. Subjects with events had larger LV mass and lower LV-GLS as compared to cases with no events. In the case of an event, a higher ratio of subjects had LV-GLS < 14.77% (Table 1).

### 3.6. Multivariable Analysis

The logistic regression model identified LV-GLS (hazard ratio [HR] = 1.68, 95% confidence interval (CI) of HR: 0.996 − 2.834, *p* = 0.05) and age (HR = 1.12, 95% CI of HR: 1.048–1.196, *p* = 0.001) as independent predictors of cardiovascular survival.

## 4. Discussion

The 3DSTE method is one of the latest echocardiographic developments which is suitable for accurate quantitative determination of the volume, deformation and rotational mechanics of the LV [8,9,10,11,12]. With the help of a virtually created 3D LV model, in addition to the LV volume and LV-EF, characteristics of LV deformation can be determined for the entire LV with global parameters as well as for each segment and region of the wall in radial, longitudinal and circumferential directions [5,8,9,10,11].

Despite the fact that LV-GLS determined with 2D-STE is part of the daily routine and is a parameter with prognostic power recommended in professional guidelines, we do not have such detailed information about LV-GLS measured with 3DSTE [13,14,15,16,17]. In addition to the above, although LV-GLS is a known prognostic parameter in a general population or in certain pathologies [13,14,15,16,17], its proven significance in a completely healthy population requires further investigation. The purpose of the present study, therefore, was to investigate the prognostic impact of 3DSTE-derived LV-GLS in a population without any symptoms and considered to be healthy, where routine examinations did not indicate the presence of any disease or pathological condition.

According to the presented findings, 3DSTE-derived LV-GLS was shown to have a significant prognostic impact on event-free survival in healthy adults. During the long-term (12-year) follow-up period, approximately 8% of healthy subjects had cardiovascular events, especially in the case of older subjects. In routine examinations, 32% of the subjects who appeared healthy had lower LV-GLS, which should alert us to early subclinical abnormalities, which may raise the possibility of an early sign of some disease and possible later development. Moreover, in these cases, dilated LV dimensions and reduced pumping function were demonstrated. In fact, 70% of the events detected during the follow-up occurred in this group. These results highlight the need for closer cardiovascular control in seemingly healthy cases where LV-EF is normal but LV-GLS is impaired.

## 5. Limitation Section

- The purpose of the present study was only to assess the prognostic significance of 3DSTE-derived LV-GLS. The evaluation of other LV volumetric or functional parameters and that of other heart chambers were not aimed to be assessed.
- A problem that still exists today is the image quality of 3DSTE being worse as compared to that of 2D echocardiography due to lower spatial and temporal resolution and in the case of older models due to the larger transducer [8,9,10,11].
- Only in the case of sinus rhythm can a 3DSTE examination be performed, which in the present study was not a problem, as we were examining only healthy subjects.
- The 3DSTE method underestimates LV volumetric parameters and LV-EDV is more affected than LV-ESV, which can explain lower LV-EF values [18]. Additionally, lower 3DSTE-derived volumes could have an impact on LV-GLS measurements as well.
- There were subjects who were missing in some experiments.

## 6. Conclusions

3DSTE-derived LV-GLS is a strong independent predictor of cardiovascular survival in healthy adults.

## Figures and Tables

**Table 1:** Clinical, echocardiographic and follow-up data of healthy adults.

	All Subjects	LV-GLS ≥ 14.77%	LV-GLS < 14.77%	No Event	Event
No. of patients	124	84 (68)	40 (32)	114 (92)	10 (8)
Males (%)	64 (52)	40 (48)	24 (60)	58 (51)	6 (60)
Age (years)	31.0 ± 11.7	29.6 ± 10.5	33.9 ± 13.6	29.4 ± 9.7	49.9 ± 16.2 †
Two-dimensional echocardiography					
LV-EDD (mm)	48.1 ± 3.7	47.9 ± 3.6	48.7 ± 3.8	48.0 ± 3.7	49.3 ± 3.0
LV-EDV (mL)	105.9 ± 21.8	102.9 ± 21.4	112.3 ± 21.5 *	105.4 ± 22.1	112.4 ± 16.8
LV-ESD (mm)	31.7 ± 3.2	31.3 ± 3.1	32.5 ± 3.4 *	31.7 ± 3.4	31.9 ± 1.4
LV-ESV (mL)	35.6 ± 8.7	33.8 ± 8.3	39.6 ± 8.4 *	35.4 ± 8.9	38.9 ± 5.0
IVS (mm)	9.0 ± 1.6	8.9 ± 1.6	9.2 ± 1.5	8.9 ± 1.6	10.1 ± 1.7 †
LV-PW (mm)	9.0 ± 1.7	9.0 ± 1.8	9.1 ± 1.4	9.0 ± 1.7	10.0 ± 1.4
LV-EF (%)	66.4 ± 5.3	67.4 ± 4.9	64.4 ± 5.6 *	66.5 ± 5.3	65.0 ± 4.3
Three-dimensional speckle-tracking echocardiography					
LV-EDV (mL)	86.1 ± 23.4	86.4 ± 24.3	85.5 ± 21.6	85.9 ± 23.3	88.4 ± 24.8
LV-EDV/BSA (mL/m^2^)	46.5 ± 13.2	46.4 ± 13.5	46.2 ± 11.0	46.0 ± 13.1	47.5 ± 13.8
LV-ESV (mL)	36.5 ± 10.0	36.2 ± 9.8	36.9 ± 10.5	36.3 ± 9.7	38.8 ± 13.2
LV-ESV/BSA (mL/m^2^)	19.8 ± 6.0	19.6 ± 6.2	20.0 ± 6.8	19.6 ± 6.3	21.0 ± 7.1
LV-EF (%)	57.9 ± 5.1	58.5 ± 4.8	56.7 ± 5.6	58.0 ± 5.0	56.4 ± 5.7
LV-mass (g)	160.2 ± 30.2	157.3 ± 29.4	166.2 ± 31.5	158.6 ± 29.2	177.5 ± 38.0 †
LV-GLS (%)	16.1 ± 2.3	17.3 ± 1.7	13.6 ± 0.9 *	16.2 ± 2.3	14.3 ± 1.5 †
Pts with LV-GLS < 14.77%	55 (44)	0 (0)	55 (100) *	33 (29)	7 (70) †
Events					
Pts with events (%)	10 (8)	3 (4)	7 (18) *	0 (0)	10 (100) †
Pts with death (%)	2 (2)	0 (0)	2 (5)	0 (0)	2 (20) †

† *p* < 0.05 vs. no events; * *p* < 0.05 vs. LV-GLS ≥ − 14.77%. Abbreviations: LV = left ventricular, EDD = end-diastolic diameter, EDV = end-diastolic volume, ESD = end-systolic diameter, ESV = end-systolic volume, IV = interventricular septum, PW = posterior wall, EF = ejection fraction, GLS = global longitudinal strain.

## Data Availability

The authors take responsibility for all aspects of the reliability and freedom from bias of the data presented and their discussed interpretation.
